# Association of Angiotensin II Type 1 Receptor Agonistic Autoantibodies With Outcomes in Patients With Acute Aortic Dissection

**DOI:** 10.1001/jamanetworkopen.2021.27587

**Published:** 2021-10-01

**Authors:** Xiao-wei Wu, Gang Li, Xiao-bin Cheng, Min Wang, Liu-lin Wang, Hai-hao Wang, Jian-ye Yang, Xing-jian Hu

**Affiliations:** 1 Department of Thoracic Surgery, Tongji Hospital, Tongji Medical College, Huazhong University of Science and Technology, Wuhan, China; 2 Emergency Department, Hubei Provincial Hospital of Traditional Chinese Medicine, Wuhan, China; 3 Hubei Province Academy of Traditional Chinese Medicine, Wuhan, China; 4 Department of Cardiothoracic and Vascular Surgery, Tongji Hospital, Tongji Medical College, Huazhong University of Science and Technology, Wuhan, China; 5 Department of Cardiovascular Surgery, Union Hospital, Tongji Medical College, Huazhong University of Science and Technology, Wuhan, China

## Abstract

**Importance:**

Angiotensin II is significantly associated with the pathogenesis of acute aortic dissection. Angiotensin II type 1 receptor agonistic autoantibodies (AT1-AAs) can mimic the effect of angiotensin II.

**Objective:**

To investigate the association between AT1-AAs and all-cause and cause-specific mortality risk in patients with acute aortic dissection.

**Design, Setting, and Participants:**

A total of 662 patients with clinically suspected aortic dissection from 3 medical centers in Wuhan, China, were enrolled in this cohort study from August 1, 2014, to July 31, 2016. Of these, 315 patients were included in the 3-year follow-up study. Follow-up was mainly performed via telephone interviews and outpatient clinic visits. Data analysis was conducted from March 1 to May 31, 2020.

**Main Outcomes and Measures:**

The primary outcomes of interest were all-cause mortality, death due to aortic dissection, and late aortic-related adverse events.

**Results:**

The full study cohort included 315 patients with AAD (mean [SD] age, 56.2 [12.7] years; 230 men [73.0%]). Ninety-two patients (29.2%) were positive for AT1-AAs. The mortality of AT1-AA–positive patients was significantly higher than that of AT1-AA–negative patients (40 [43.5%] vs 37 [16.6%]; *P* < .001). The mortality risk in AT1-AA–positive patients was always significantly higher than that in AT1-AA–negative patients in patients with both type A and type B dissection. Multivariable analysis showed that the risk of AT1-AA–positive patients for type A dissection was significantly higher than that of AT1-AA–negative patients (odds ratio [OR], 1.88; 95% CI, 1.12-3.13; *P* = .02). The Cox proportional hazards regression model showed a significant increase of all-cause mortality risk (OR, 2.27; 95% CI, 1.44-3.57; *P* < .001) and late aortic-related adverse events (OR, 1.58; 95% CI, 1.06-2.36; *P* = .03) among AT1-AA–positive patients during the follow-up period compared with AT1-AA–negative patients.

**Conclusions and Relevance:**

This cohort study first detected AT1-AAs in patients with acute aortic dissection. The presence of AT1-AAs was associated with significantly higher all-cause and cause-specific mortality during a follow-up period of 3 years. The antibodies may be a risk factor for aortic dissection.

## Key Points

**Question:**

Is there an association between angiotensin II type 1 receptor agonistic autoantibodies (AT1-AAs) and all-cause mortality among patients with aortic dissection?

**Findings:**

This population-based cohort study of 315 adults with acute aortic dissection suggests that AT1-AAs are associated with an increased risk of all-cause mortality, aortic-related death, and late aortic-related adverse events.

**Meaning:**

Patients with acute aortic dissection who are positive for AT1-AAs may have a higher risk of mortality.

## Teaser

This population-based cohort study assessed whether expression of angiotensin II type 1 receptor agonistic autoantibodies is associated with all-cause and cause-specific mortality in patients with aortic dissection.

## Introduction

In aortic dissection, the innermost layer of the aorta is torn owing to various reasons, including immunity, inflammation, genetic factors, hypertension, and oxidative stress.^1^ This condition can lead to aortic rupture and eventual death. At present, the mechanisms that cause aortic dissection remain unclear. Studies have shown that angiotensin II is significantly associated with the pathogenesis of aortic dissection.^2,3^ In an animal model study, Ju et al^4^ continuously pumped angiotensin II into the abdominal cavity of C57BL/6 mice and found that aortic dissection formed only 1 week later. Angiotensin II receptor blockers (ARBs) were able to reduce aortic aneurysm expansion more effectively than angiotensin-converting enzyme inhibitors, suggesting that the angiotensin II type 1 receptor (AT1-R) plays an important role in the development of aortic dissection.^5^

Khan et al^6^ found that 62% to 78% of patients with aortic dissection had a history of hypertension. More than 40% of patients with refractory hypertension have AT1-R agonistic autoantibodies (AT1-AAs) that can partially mimic the effect of angiotensin II.^7^ A previous study by Li et al^8^ found that the presence of AT1-AAs is significantly associated with the progression of arteriosclerosis in a population with normal blood pressure. The AT1-AAs can promote the expression of endothelin 1 and injure the arterial intima by binding to the AT1-R.^9^ Angiotensin II type 1 receptor agonistic autoantibodies can also promote the proliferation of vascular smooth muscle cells by activating the ERK1/2 pathway and inducing aortic wall stiffening.^10^ Angiotensin II also stimulates the production of matrix metalloproteinases (MMPs) through various pathways.^11^ Matrix metalloproteinases are deeply involved in the pathogenesis of aortic dissection. Whether AT1-AAs are expressed in patients with aortic dissection has not been confirmed, to the best of our knowledge. Therefore, the purpose of this study was to clarify the expression of AT1-AAs in patients with aortic dissection and to analyze the association between AT1-AAs and the outcomes of patients with the condition.

## Methods

### Study Participants

Based on the aortic dissection detection (ADD) risk score,^12^ a total of 662 patients with clinically suspected aortic dissection from 3 medical centers were enrolled in this cohort study from August 1, 2014, to July 31, 2016. A total of 410 patients were diagnosed with aortic dissection after appropriate examinations and signed the informed consent document, of whom 315 were included in the final analysis after 3 years of follow-up. Baseline demographic and clinical data were collected from the hospital computerized patient registry and electronic medical record. Clinical variables were recorded on a standardized form that included information on patient history, demographics, physical examination findings, imaging study results, clinical presentations, details of medical and surgical treatment, and patient outcomes, including mortality. Blood samples were collected after patients provided written informed consent. The end point event was 3-year mortality. Follow-up was mainly performed via telephone interviews and outpatient clinic visits. A flowchart of participant selection is shown in eFigure 1 in the Supplement. This cohort study was conducted in accordance with the Declaration of Helsinki,^13^ and the study protocol was approved by the Institutional Ethics Committee of Tongji Hospital, Tongji Medical College, Huazhong University of Science and Technology, Wuhan, China. Written informed consent was obtained from all participants. This study followed the Strengthening the Reporting of Observational Studies in Epidemiology (STROBE) reporting guideline.

Patients diagnosed with aortic dissection had undergone aortic computed tomographic angiography examination and transthoracic echocardiography.^14^ Inclusion criteria were age 18 to 80 years, an interval from onset to admission within 6 hours, and intermediate and high ADD risk score. The ADD risk score was calculated based on the number of risk categories (high-risk predisposing conditions, high-risk pain features, and high-risk examination features) in which patients met criteria.^12^ An ADD score of 1 was defined as intermediate risk and an ADD score of 2 or 3 was defined as high risk.^12^ Exclusion criteria were significant changes in electrocardiographic findings, acute myocardial infarction, acute pericardial tamponade, acute myocarditis, and a family history of Marfan syndrome.

### Definitions

In this study, an early event was defined as occurring within 30 days after the diagnosis. A late event was defined as one associated with the dissection that occurred more than 30 days after the diagnosis. Aortic-related adverse events included rupture, aortic enlargement (>60 mm), retrograde type A aortic dissection, ulcerlike projection (defined as a localized blood-filled pouch protruding from the true lumen into the thrombosed false lumen of the aorta), endoleak, and stent graft–induced new entry. Late death was defined as any death that occurred more than 30 days after the diagnosis of dissection. Aortic-related death included operative deaths and deaths that occurred as a result of aortic-related adverse events. Patients’ electronic medical records were used to determine the cause of death.

### Detection of Serum MMP-9 Level and Biochemical Blood Indexes

For measurement of MMP-9 levels, 3 to 5 mL of venous blood was drawn within 1 hour after aortic dissection was diagnosed if the patients or their family members had signed an informed consent document. Sandwich enzyme-linked immunosorbent assay (RayBiotech Life, Inc) was used to test for MMP-9 according to the manufacturer’s instructions. Other analyte levels, such as cardiac troponin T, high-sensitivity C-reactive protein, interleukin 6 (IL-6), creatinine, and serum urea nitrogen, were measured at the testing center of the affiliated hospital according to standard methods.

### Serum AT1-AA Measurement

The presence of AT1-AAs was evaluated using an AT1-R enzyme-linked immunosorbent assay (Lot 6 kit; One Lambda). During collection, venous blood was drawn into a vacuum tube. The samples were centrifuged at 1000*g* for 15 minutes. Serum was collected and stored at −80 °C until the date of measurement. Standard and diluted (1:100) samples were added into the wells and incubated for 2 hours at 2 °C to 8 °C. After the washing steps, anti–AT1-R antibody was detected with peroxidase-labeled anti–human IgG antibody (1:100) followed by color development with 3,30,5,50-tetramethylbenzidine solution. Measurements were performed at 450 nm, with a correction wavelength of 630 nm. The detection range for the test was greater than 2.5 U/mL, with intermediate positive values set at 10 to 17 U/mL, strong positive value set at greater than 17 U/mL, and negative value set at less than 10 U/mL according to previous studies and manufacturer recommendations.^15,16^

### Statistical Analysis

Demographic and medical data meeting normal distribution requirements are presented as mean (SD). Data with a skewed distribution are presented as median (IQR). After adjusting for age and sex, a general linear model was used to analyze the differences in maximum aortic diameter (MAD) and MMP-9 level between autoantibody-positive and -negative patients. Levels of AT1-AAs and MMP-9 were logarithmically transformed to approximate a normal distribution. After adjusting for age, sex, and dissection type, partial correlation analysis was used to study the correlation between log(AT1-AA) and log(MMP-9) or log(MAD). We included sex and age at baseline as potential confounders in the model. In addition, the fully adjusted model included sex, age, hypertension, diabetes, MAD, treatment method, and levels of MMP-9, serum urea nitrogen, D-dimer, cardiac troponin T, high-sensitivity C-reactive protein, and IL-6. These covariates were also included as time-varying covariates in time-dependent Cox proportional hazards regression analyses. The rationale for inclusion as covariates in models was that these variables were associated with exposures and outcomes. After adjusting for related variables, multivariable logistic regression was used to analyze the association between autoantibodies with a risk of death due to type A and type B dissection. Autoantibody association with MAD was also analyzed using multivariable logistic regression. We used Cox proportional hazards regression to estimate the hazard ratio for death in autoantibody-positive patients relative to autoantibody-negative patients. All statistical analyses were performed using SPSS software, version 22.0 (SPSS Inc), and 2-sided *P* < .05 indicated statistical significance. Data analysis was performed from March 1 to May 31, 2020.

## Results

The study population included 410 patients (mean [SD] age, 57.3 [11.5] years) with a diagnosis of acute aortic dissection who signed the informed consent document. Of those, 315 patients (76.8%; mean [SD] age, 56.2 [12.7] years) had complete information available for all follow-up and late adverse event records (46 patients were lost to follow-up and 49 did not have complete outpatient follow-up records [eFigure 1 in the Supplement]). The cohort included 230 men (73.0%) and 85 women (27.0%). A total of 108 patients had type A dissection and 207 had type B dissection. Two hundred thirty-four patients (74.3%) had a history of hypertension. Among the full cohort of 315 patients, 92 (29.2%) had positive findings for AT1-AAs. After a mean (SD) follow-up of 36.0 (12.9) months, a total of 77 patients died. The mortality of AT1-AA–positive patients was significantly higher than that of AT1-AA–negative patients (40 [43.5%] vs 37 [16.6%]; *P* < .001). In addition, the proportion of patients with type A dissection among AT1-AA–positive patients was significantly higher than that in AT1-AA–negative patients (42 [45.7%] vs 66 [29.6%]; *P* = .02). The proportion of AT1-AA–positive results was higher in patients with type A dissection (43 [39.8%] vs 55 [26.7%]; *P* = .03). Patients positive for AT1-AAs had a significantly greater MAD (mean [SD], 45.8 [7.0] vs 44.0 [6.1] mm; *P* = .02), higher levels of MMP-9 (median, 43.9 [IQR, 38.4-50.3] vs 36.4 [IQR, 30.9-39.7] ng/mL; *P* < .001) and serum IL-6 (mean [SD], 13.43 [6.66] vs 8.07 [4.99] pg/mL; *P* < .001), and higher proportion of grade 3 hypertension (35 [38.0%] vs 48 [21.5%]; *P* = .002) compared with patients negative for AT1-AA (Table 1). These patients had 14 outpatient visits with blood pressure recorded. The results showed that there was no significant difference in systolic or diastolic blood pressure between the 2 groups (eFigure 2 in the Supplement). General linear model analysis showed that after adjusting for age and sex, MAD (mean [SD], 44.9 [6.5] vs 43.1 [4.4] mm; *P* = .02) and serum MMP-9 level (mean [SD], 45.1 [8.6] vs 36.2 [5.7] ng/mL; *P* < .001) in patients positive for AT1-AA were still significantly higher than in patients negative for AT1-AA (eFigure 3 in the Supplement). Partial correlation analysis showed that after adjusting for age, sex, and dissection type, the level of AT1-AAs in the peripheral circulation was significantly correlated with MAD (*r* = 0.159; *P* = .006) and MMP-9 level (*r* = 0.524; *P* = .005) (eFigure 3 in the Supplement).

**Table 1.** Clinical and Laboratory Data for Patients With Acute Aortic Dissection^a^

Clinical variable	Patient group		*P* value	Patient group		*P* value	Total (N = 315)
	AT1-AA–negative (n = 223)	AT1-AA–positive (n = 92)		Type A dissection (n = 108)	Type B dissection (n = 207)		
Age, mean (SD), y	55.5 (12.2)	57.6 (13.7)	.18	54.6 (13.5)	57.0 (12.2)	.12	56.2 (12.7)
Sex							
Men	160 (71.7)	70 (76.1)	.77	74 (68.5)	156 (75.4)	.12	230 (73.0)
Women	63 (28.3)	22 (23.9)	.49	34 (31.5)	51 (24.6)	.23	85 (27.0)
BMI, mean (SD)	26.2 (2.3)	26.5 (2.5)	.20	26.6 (2.1)	26.1 (2.4)	.09	26.3 (2.4)
Death	37 (16.6)	40 (43.5)	<.001	44 (40.7)	33 (15.9)	<.001	77 (24.4)
Hypertension	164 (73.5)	70 (76.1)	.81	85 (78.7)	149 (72.0)	.12	234 (74.3)
Grade 3 hypertension	48 (21.5)	35 (38.0)	.002	39 (36.1)	44 (21.3)	.004	83 (26.3)
History of diabetes	38 (17.0)	14 (15.2)	.47	17 (15.7)	35 (16.9)	.50	52 (16.5)
Family income, ¥/y							
<50 000	27 (12.1)	17 (18.5)	.23	19 (17.6)	25 (12.1)	.07	44 (14.0)
50 000-150 000	151 (67.7)	54 (58.7)		61 (56.5)	144 (69.6)		205 (65.1)
>150 000	45 (20.2)	21 (22.8)		28 (25.9)	38 (18.4)		66 (21.0)
Smoking	139 (62.3)	65 (70.7)	.20	75 (69.4)	129 (62.3)	.22	204 (64.8)
Hospitalization time, mean (SD), d	18.5 (12.3)	18.9 (14.4)	.84	28.8 (14.6)	13.5 (8.3)	<.001	18.7 (13.0)
Stanford A dissection	66 (29.6)	42 (45.7)	.02	NA	NA	NA	108 (34.3)
Surgical or interventional treatment	119 (53.4)	44 (47.8)	.09	41 (38.0)	122 (58.9)	<.001	163 (51.7)
ARB/ACEI	60 (26.9)	29 (31.5)	.24	32 (29.6)	57 (27.5)	.40	89 (28.3)
CCB	76 (34.1)	23 (25.0)	.07	28 (25.9)	71 (34.3)	.08	99 (31.4)
β-blocker	35 (15.7)	18 (19.6)	.25	19 (17.6)	34 (16.4)	.45	53 (16.8)
Statin	92 (41.3)	35 (38.0)	.35	48 (44.4)	79 (38.2)	.17	127 (40.3)
Aspirin	67 (30.0)	25 (27.2)	.36	28 (25.9)	64 (30.9)	.21	92 (29.2)
MAD, mean (SD), mm	44.0 (6.1)	45.8 (7.0)	.02	46.5 (6.4)	43.5 (6.2)	<.001	44.5 (6.4)
AT1-AA level, mean (SD), U/mL	7.1 (2.2)	17.2 (6.3)	<.001	12.2 (7.5)	9.2 (5.0)	<.001	10.2 (6.1)
Admission blood pressure, mean (SD), mm Hg							
Systolic	147.7 (27.5)	150.6 (27.4)	.39	148.6 (29.7)	148.8 (26.3)	.98	148.6 (27.5)
Diastolic	86.0 (17.6)	87.3 (16.8)	.54	84.0 (19.4)	87.6 (16.1)	.08	86.4 (17.3)
Heart rate, mean (SD), bpm	83.3 (14.1)	84.0 (15.7)	.69	83.9 (15.6)	83.3 (14.1)	.72	83.5 (14.6)
hs-CRP level, mean (SD), mg/dL	1.07 (0.90)	1.05 (0.77)	.87	1.23 (0.94)	0.98 (0.80)	.02	1.07 (0.86)
IL-6 level, mean (SD), pg/mL	8.07 (4.99)	13.43 (6.66)	<.001	10.89 (6.29)	9.17 (5.93)	.02	9.75 (6.09)
Uric acid level, mean (SD), mg/dL	5.44 (1.85)	5.41 (1.81)	.88	5.34 (1.61)	5.48 (1.94)	.52	5.43 (1.84)
Creatinine level, mean (SD), mg/dL	1.2 (0.6)	1.3 (0.7)	.13	1.3 (0.6)	1.1 (0.6)	.06	1.2 (0.6)
SUN, mean (SD), mg/dL	20.2 (10.1)	22.4 (12.3)	.09	23.2 (11.8)	19.6 (10.4)	.004	20.7 (10.9)
MMP-9 level, median (IQR), ng/mL	36.4 (30.9-39.7)	43.9 (38.4-50.3)	<.001	37 (33-42)	37 (33-43)	.40	36.9 (33.4-43.5)
D-dimer level, median (IQR), μg/mL	50.9 (29.3-98.5)	57.8 (45.1-93.3)	.93	62.3 (39.7-130.2)	50.8 (29.4-85.0)	.001	54.9 (33.2-97.4)
cTnT level, mean (SD), ng/mL	0.105 (0.290)	0.101 (0.183)	.90	0.203 (0.398)	0.053 (0.124)	<.001	0.104 (0.262)

Abbreviations: ACEI, angiotensin-converting enzyme inhibitor; ARB, angiotensin II receptor blocker; AT1-AA, angiotensin II type 1 receptor agonistic autoantibody; BMI, body mass index (calculated as weight in kilograms divided by height in meters squared); CCB, calcium channel blocker; cTnT, cardiac troponin T; hs-CRP, high-sensitivity C-reactive protein; IL-6, interleukin-6; MAD, maximum aortic diameter; MMP-9, matrix metalloproteinase 9; NA, not applicable; SUN, serum urea nitrogen.

SI conversion factors: To convert creatinine to μmol/L, multiply by 88.4; cTnT to μg/L, multiply by 1.0; D-dimer to nmol/L, multiply by 5.476; hs-CRP to mg/L, multiply by 10; SUN to mmol/L, multiply by 0.357; and uric acid to μmol/L, multiply by 59.5.

^a^ Unless otherwise indicated, data are expressed as number (%) of patients. Percentages have been rounded and may not total 100.

Details of early and late events are listed in Table 2. In the AT1-AA–negative group, 6 early major complications were observed, and in the AT1-AA–positive group, 4 early major complications were observed, with no significant difference between the 2 groups (*P* = .33). In the AT1-AA–positive group, the 30-day mortality (26 [28.3%] vs 27 [12.1%]) and late mortality (14 [15.2%] vs 10 [4.5%]) were significantly higher than in the AT1-AA–negative group (Table 2). A total of 99 patients had late events. The late event rate was significantly higher in the AT1-AA–positive group (38 [41.3%]) compared with the AT1-AA–negative group (61 [27.4%]) (*P* = .01) (Table 2). Aortic enlargement (n = 47) was the most common late event in both groups. Among the 77 deaths that were reported, 45 were aortic-related deaths, 20 were aortic-unrelated deaths, and the cause was unknown in 12. A total of 24 late deaths were reported, including 17 aortic-related late deaths, 14 of which were due to a ruptured false lumen in the descending aorta and the remaining 3 to retrograde type A aortic dissection. The aortic-related and aortic-unrelated mortality of the AT1-AA–positive group (21 [22.8%] and 12 [13.0%], respectively) were significantly higher than those of the AT1-AA–negative group (24 [10.8%; *P* = .006] and 8 [3.6%; *P* = .003], respectively).

**Table 2.** Early and Late Outcomes in AT1-AA–Negative and –Positive Patients^a^

Outcome	Patient group		*P* value
	AT1-AA–negative (n = 223)	AT1-AA–positive (n = 92)	
Early major complication	6 (2.7)	4 (4.3)	
Organ failure	3 (0.1)	1 (1.1)	.33
Stroke	2 (0.9)	1 (1.1)	
Rupture	1 (0.4)	2 (2.2)	
Treatment methods			
Open surgery	25 (11.2)	16 (17.4)	.14
TEVAR	94 (42.2)	28 (30.4)	.06
Medical treatment	104 (46.6)	48 (52.2)	.22
30-d Death	27 (12.1)	26 (28.3)	.001
Late death	10 (4.5)	14 (15.2)	.002
Late events	61 (27.4)	38 (41.3)	
Type I endoleak	8 (3.6)	3 (3.3)	.01
Rupture	11 (4.9)	5 (5.4)	
Retrograde type A aortic dissection	5 (2.2)	2 (2.2)	
Aortic enlargement	27 (12.1)	20 (21.7)	
Ulcerlike projection	10 (4.5)	8 (8.7)	
Cause of death			
Aortic-related	24 (10.8)	21 (22.8)	.006
Aortic-unrelated	8 (3.6)	12 (13.0)	.003
Unknown	5 (2.2)	7 (7.6)	.03

Abbreviations: AT1-AA, angiotensin II type 1 receptor agonistic autoantibody; TEVAR, thoracic endovascular aortic repair.

^a^ Data are presented as number (%) of patients.

Multivariable logistic regression analysis was performed to clarify the association between AT1-AAs and the risk of death in 108 patients with type A dissection. Before (odds ratio [OR], 2.50; 95% CI, 1.11-5.61; *P* = .03) and after (OR, 5.08; 95% CI, 1.47-17.65; *P* = .01) adjusting for the confounding factor, the risk of death in AT1-AA–positive patients was significantly higher than that in AT1-AA–negative patients (Table 3). Similar multivariable logistic regression analysis was performed in 207 patients with type B dissection. Regardless of the adjustment for the confounding factor, the risk of death in AT1-AA–positive patients was significantly higher than that in AT1-AA–negative patients and exhibited an increasing trend (unadjusted OR, 3.79 [95% CI, 1.75-8.22; *P* = .001]; adjusted OR, 4.63 [95% CI, 1.41-14.58; *P* = .01]) (Table 3). Although the death risk in patients with type B dissection was significantly lower than that in patients with type A dissection, the death risk in AT1-AA–positive patients was always significantly higher than that in AT1-AA–negative patients in patients with both type A and type B dissection.

**Table 3.** Logistic Regression Analysis of AT1-AAs and Mortality in Patients With Acute Aortic Dissection

	β Coefficient (SE)	OR (95% CI)	*P* value
**Type A acute aortic dissection (n = 108)**			
Unadjusted	0.914 (0.414)	2.50 (1.11-5.61)	.03
Model 1^a^	0.986 (0.460)	2.68 (1.09-6.60)	.03
Model 2^b^	1.018 (0.483)	2.77 (1.08-7.13)	.04
Model 3^c^	1.626 (0.635)	5.08 (1.47-17.65)	.01
**Type B acute aortic dissection (n = 207)**			
Unadjusted	1.333 (0.395)	3.79 (1.75-8.22)	.001
Model 1^a^	1.236 (0.406)	3.44 (1.55-7.64)	.002
Model 2^b^	1.108 (0.468)	3.03 (1.21-7.58)	.02
Model 3^c^	1.521 (0.604)	4.63 (1.41-14.58)	.01

Abbreviations: AT1-AAs, angiotensin II type 1 receptor agonistic autoantibodies; OR, odds ratio.

^a^ Adjusted for age, sex, hypertension, and diabetes.

^b^ Adjusted for covariates in model 1, maximum aortic diameter, and surgical or interventional treatment.

^c^ Adjusted for covariates in model 2 and levels of matrix metalloproteinase 9, serum urea nitrogen, D-dimer, cardiac troponin T, high-sensitivity C-reactive protein, and interleukin 6.

Risk factors for type A dissection were identified using logistic regression. Univariate analysis showed that the risk of AT1-AA–positive patients for type A dissection was significantly higher than that of AT1-AA–negative patients (OR, 1.81; 95% CI, 1.10-3.00; *P* = .02). Multivariable analysis after adjusting for age, sex, hypertension status, and diabetes status still showed a significantly increased risk for type A dissection in AT1-AA–positive patients relative to AT1-AA–negative patients (OR, 1.88; 95% CI, 1.12-3.13; *P* = .02).

Multivariable logistic regression analysis results suggested that the type of aortic dissection is closely associated with MAD (β coefficient, 2.968; 95% CI, 1.454-4.480; *P* < .001) (eTable in the Supplement). After adjusting for age, sex, hypertension, and dissection type, AT1-AA status could be used to estimate the MAD (β = 0.083; 95% CI, 0.024-0.142; *P* = .006) (eTable in the Supplement).

**Figure 1.**
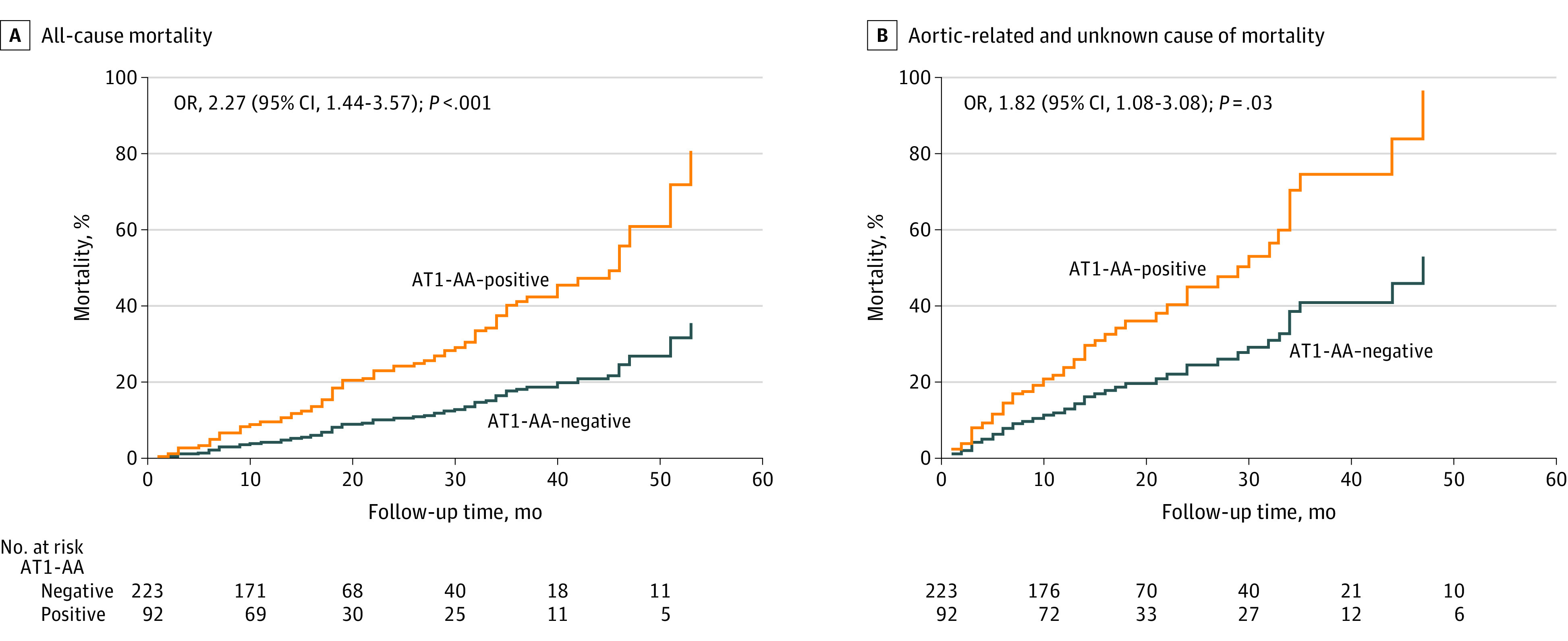
Cox Proportional Hazards Regression Analysis of Mortality Between Autoantibody-Positive and -Negative Patients Data were adjusted for age, sex, hypertension, diabetes, maximum aortic diameter, treatment method, and levels of matrix metalloproteinase 9, serum urea nitrogen, D-dimer, cardiac troponin T, high-sensitivity C-reactive protein, and interleukin 6. AT1-AA indicates angiotensin II type 1 receptor agonistic autoantibody.

**Figure 2.**
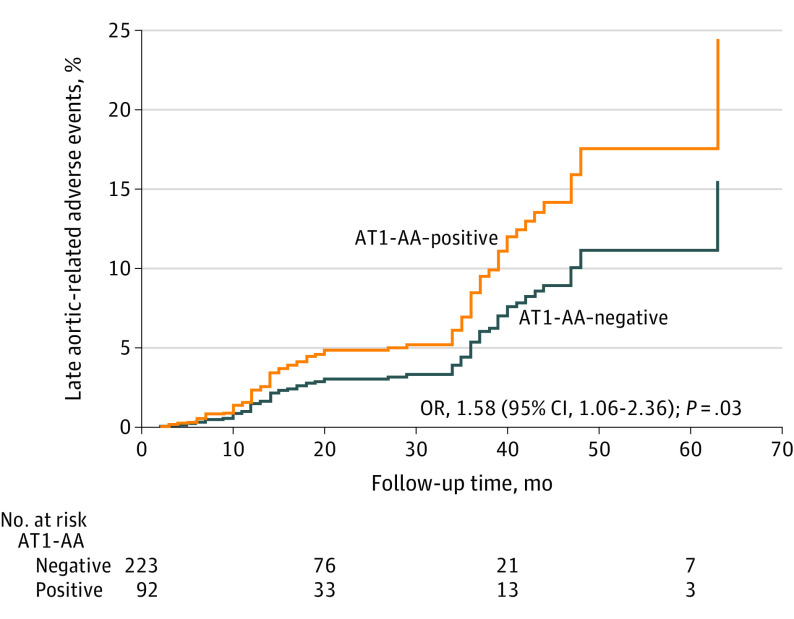
Late Aortic-Related Adverse Events Between Autoantibody-Positive and -Negative Patients Data were adjusted for age, sex, hypertension, diabetes, maximum aortic diameter, treatment method, and levels of matrix metalloproteinase 9, serum urea nitrogen, D-dimer, cardiac troponin T, high-sensitivity C-reactive protein, and interleukin 6. AT1-AA indicates angiotensin II type 1 receptor agonistic autoantibody.

A Cox proportional hazards regression model showed that in multivariable analyses after adjusting for confounding factors, risk of all-cause death (OR, 2.27; 95% CI, 1.44-3.57; *P* < .001) (Figure 1A) and aortic-related and unknown death risk (OR, 1.82; 95% CI, 1.08-3.08; *P* = .03) (Figure 1B) among AT1-AA–positive patients during the follow-up period increased significantly relative to that of AT1-AA–negative patients. The AT1-AA–positive patients experienced significantly more late aortic-related adverse events compared with AT1-AA–negative patients (OR, 1.58; 95% CI, 1.06-2.36; *P* = .03) (Figure 2).

## Discussion

To our knowledge, this study is the first to detect AT1-AAs in patients with acute aortic dissection. Ninety-two of the patients in this study were positive for AT1-AAs. Mortality in AT1-AA–positive patients was significantly higher than that in AT1-AA–negative patients. The AT1-AA–positive patients had a significantly larger MAD and higher MMP-9 and IL-6 serum levels than AT1-AA–negative patients. During follow-up, the death risk and late aortic-related adverse events were significantly increased in AT1-AA–positive patients compared with AT1-AA–negative patients.

Aortic dissection is an extremely dangerous condition, with an annual incidence ranging from 2.6 to 3.5 per 100 000 people and a male-to-female ratio of 3:2. The incidence is still increasing, and the death rates 6 and 24 hours after onset in untreated patients are 54% and 76%, respectively.^14^ Hypertension is the most common risk factor for aortic dissection. In addition to hypertension, the role of immune and inflammatory factors in dissection formation has also received increasing attention.^17^ Angiotensin II type 1 receptor agonistic autoantibodies were first found in patients with preeclampsia.^18^ However, it was later discovered that AT1-AA expression was not rare in patients with primary hypertension. Liao et al^7^ reported that AT1-AAs were present in 43% of patients with refractory hypertension and 10.4% of those with nonrefractory hypertension. In the present study, 74.3% of patients with aortic dissection had a history of hypertension, whereas 29.2% of patients were AT1-AA–positive, particularly those with type A dissection and a higher positivity rate. Therefore, the presence of AT1-AAs is not a rare phenomenon in patients with aortic dissection.

The findings of the present study suggest a strong association between AT1-AAs and MMP-9 levels in patients with acute aortic dissection, because the levels of MMP-9 in AT1-AA–positive patients increased significantly. After adjusting for age and sex, the association between AT1-AAs and MMP-9 levels was still significant. These findings are consistent with those of Kurihara et al,^19^ who found that MMP-9 and angiotensin II levels increased significantly in patients with acute aortic dissection. An animal model showed that MMP-9 may promote rupture of acute aortic dissection via in situ stimulation of transforming growth factor-β expression.^20^ Recent studies show that an increase of MMP levels in the aortic wall of patients with aortic dissection can induce cell necrosis and degradation of the extracellular matrix, which are significantly associated with progression of aortic dissection.^21^ Angiotensin II can stimulate MMP secretion by neutrophils and smooth muscle cells in the aortic wall via multiple pathways.^22,23^ The main pathological role of the antibodies is to mimic angiotensin II. Compared with angiontensin II, receptor desensitization is more difficult via binding of AT1-AAs to AT1-R, so that AT1-AAs can impose a sustained pathological stimulating effect on AT1-R.^24^ In this mechanism, the 2 antibody arms simultaneously bind to AT1-R, which causes the receptor to cross-link and stabilizes receptor conformation.^25^ Circulating antibodies attach to the aortic wall and bind to AT1-R to continuously stimulate the production of MMPs.

A study by Abadir et al^26^ in 255 adults found that a high level of AT1-AAs was associated with a higher level of inflammatory cytokines, weaker grip strength, slower walking speed, weakness, more falls, and increased mortality and that treatment with ARBs can reduce the injury associated with high levels of AT1-AAs. To our knowledge, the present study is the first to detect antibodies in patients with acute aortic dissection, as well as the first to examine the association between the antibodies and patient prognosis. Neoantigens are processed by antigen-presenting cells (eg, dendritic cells) and are presented to helper T cells, which in turn stimulate B cells to produce AT1-AAs.^27^ After the antibodies in the peripheral circulation attached to the aortic wall by binding to AT1-R, AT1-AAs led to an imbalance in the local renin-angiotensin system and activation of multiple pathways, further inducing inflammatory factor release, endometrial damage, and dysfunction of vascular smooth muscle cells, eventually causing remodeling of the aortic wall or accelerating its tearing. Wei et al^28^ suggested that patients with AT1-AA–positive hypertension treated with ARBs have a better prognosis than patients treated with angiotensin-converting enzyme inhibitors.^28^

Li et al^29^ also found that AT1-AAs regulate the expression of inflammation-related genes via the nuclear factor κB pathway by acting on AT1-R in endothelial cells. The upregulation of vascular cell adhesion protein 1, intercellular adhesion molecule 1, monocyte chemoattractant protein 1, and IL-6 leads to endothelial dysfunction, monocyte recruitment, and inflammatory response. Angiotensin II type 1 receptor agonistic autoantibodies can interact with endogenous angiotensin II, significantly increase endothelin 1 and reactive oxygen species levels, and aggravate pathological changes in renal vascular endothelial cells.^30^ Studies have found that AT1-AAs can activate the reduced form of nicotinamide adenine dinucleotide phosphate oxidase in preeclampsia and placenta vascular smooth muscle cells.^31^ After activation, the reduced form of nicotinamide adenine dinucleotide phosphate oxidase can generate reactive oxygen species, causing damage and vascular smooth muscle cell apoptosis. The incidence of type A dissection and MAD in AT1-AA–positive patients were greater than in AT1-AA–negative patients, implying that continuous antibody expression may induce aortic remodeling. Moreover, after adjusting for age, sex, hypertension, and type of acute aortic dissection, the antibody was still significantly associated with MAD. It is suggested that AT1-AA–associated aortic remodeling may occur independently of blood pressure.

In the present study, AT1-AA–positive patients had a higher risk of unrelated aortic death. Angiotensin II type 1 receptor agonistic autoantibodies were not only associated with aortic dissection, but also with malignant hypertension, preeclampsia, aortic stiffness, and inflammatory factors. Stimulatory AT1-AAs may increase inflammatory burden, weakness, and frequency of falls, potentially worsening the condition and increasing mortality.^26^ In addition, AT1-AAs have been implicated in the pathology of congestive heart failure, leading to worse allograft outcome.^32,33^ In a previous study,^8^ AT1-AAs were identified in individuals with normal blood pressure who were followed up for 5 years. The study found that AT1-AAs were independent risk factors for progression of aortic stiffness in individuals with normal blood pressure.^8^ Aortic stiffness is a risk factor for cardiovascular and cerebrovascular diseases. The higher risk of unrelated aortic death in AT1-AA–positive patients with acute aortic dissection may be related to factors such as antibody-stimulating inflammatory factors, which can lead to worsening of the condition, aortic stiffness, and increased blood pressure. Abadir et al^26^ demonstrated that chronic treatment with ARB attenuated the AT1-AA association with decline in grip strength and increased mortality. Patients with acute aortic dissection who were positive for AT1-AAs preferred ARB drugs for antihypertensive treatment, which may further reduce the mortality risk compared with other drugs.

### Limitations

Some limitations were present in the study, including small sample size, single ethnicity constitution, and short follow-up time. Dynamic changes in antibody titer were not observed. The main contribution of the study was detecting the presence of AT1-AAs in the peripheral circulation for the first time, to our knowledge.

## Conclusions

In this cohort study, we detected AT1-AAs in the peripheral circulation of patients with acute aortic dissection and found that AT1-AAs were associated with significantly higher all-cause and cause-specific mortality during a 3-year follow-up period. The antibodies may be a risk factor for aortic dissection. More studies are required to confirm the association between the antibodies and acute aortic dissection.

## Notes

Supplement. **eFigure 1.** Study Flowchart **eFigure 2.** Blood Pressure of Antibody-Positive and -Negative Patients During Follow-up **eFigure 3.** Differences and Correlations Between MMP-9, MAD, and AT1-AA **eTable.** Multivariable Logistic Regression for Antibody Associated With MAD
