# Supplementary material for: Association of Angiotensin II Type 1 Receptor Agonistic Autoantibodies With Outcomes in Patients With Acute Aortic Dissection
Source: JAMA Netw Open. 2021 Oct 1;4(10):e2127587. doi: 10.1001/jamanetworkopen.2021.27587 (PMC8486983; doi:10.1001/jamanetworkopen.2021.27587)
Supplement: Supplement. — eFigure 1. Study Flowchart eFigure 2. Blood Pressure of Antibody-Positive and -Negative Patients During Follow-up eFigure 3. Differences and Correlations Between MMP-9, MAD, and AT1-AA eTable. Multivariable Logistic Regression for Antibody Associated With MAD [file jamanetwopen-e2127587-s001.pdf]

Supplementary Online Content

Wu XW, Li G, Cheng XB, et al. Association of angiotensin II type 1 receptor agonistic autoantibodies with outcomes in patients with acute aortic dissection. *JAMA Netw Open*. 2021;4(10):e2127587. doi:10.1001/jamanetworkopen.2021.27587

eFigure 1. Study Flowchart
eFigure 2. Blood Pressure of Antibody-Positive and -Negative Patients During Follow-up
eFigure 3. Differences and Correlations Between MMP-9, MAD, and AT1-AA
eTable. Multivariable Logistic Regression for Antibody Associated With MAD

This supplementary material has been provided by the authors to give readers additional information about their work.

© 2021 Wu X et al. *JAMA Network Open*.

eFigure 1. Study Flowchart

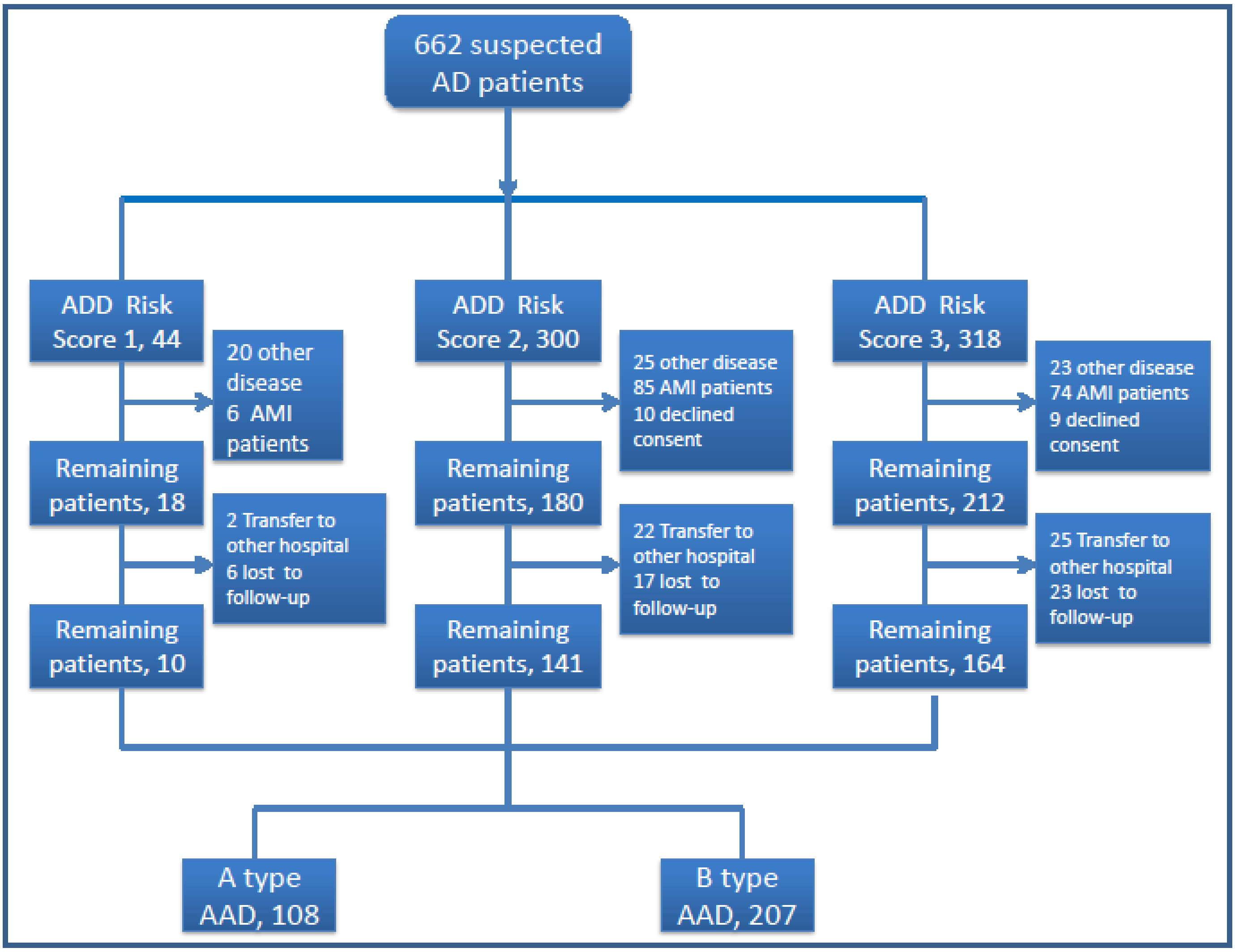

© 2021 Wu X et al. *JAMA Network Open*.

eFigure 2. Blood Pressure of Antibody-Positive and -Negative Patients During Follow-up

A

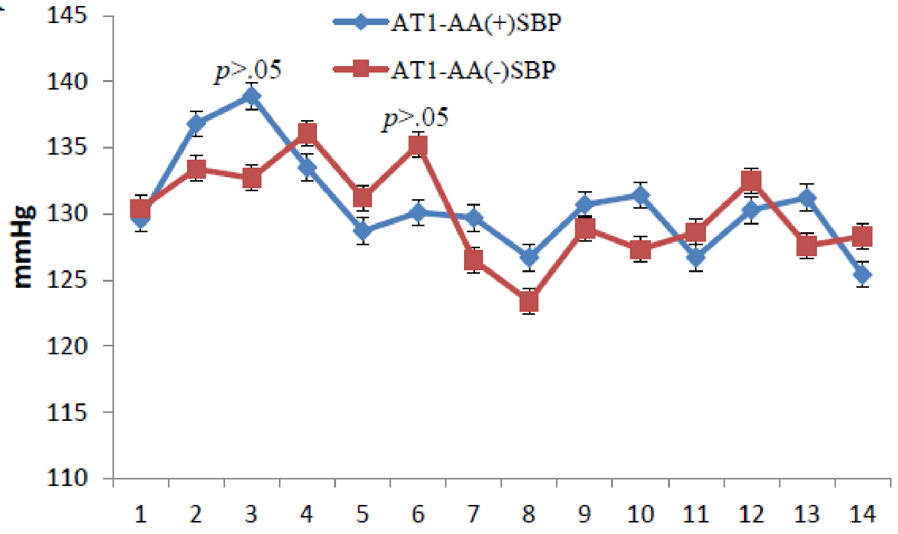

B

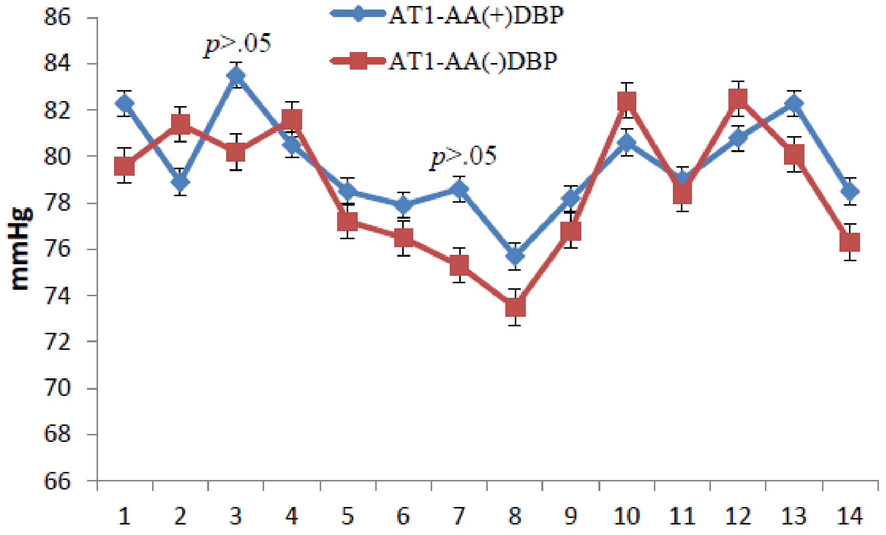

© 2021 Wu X et al. *JAMA Network Open*.

eFigure 3. Differences and Correlations Between MMP-9, MAD, and AT1-AA

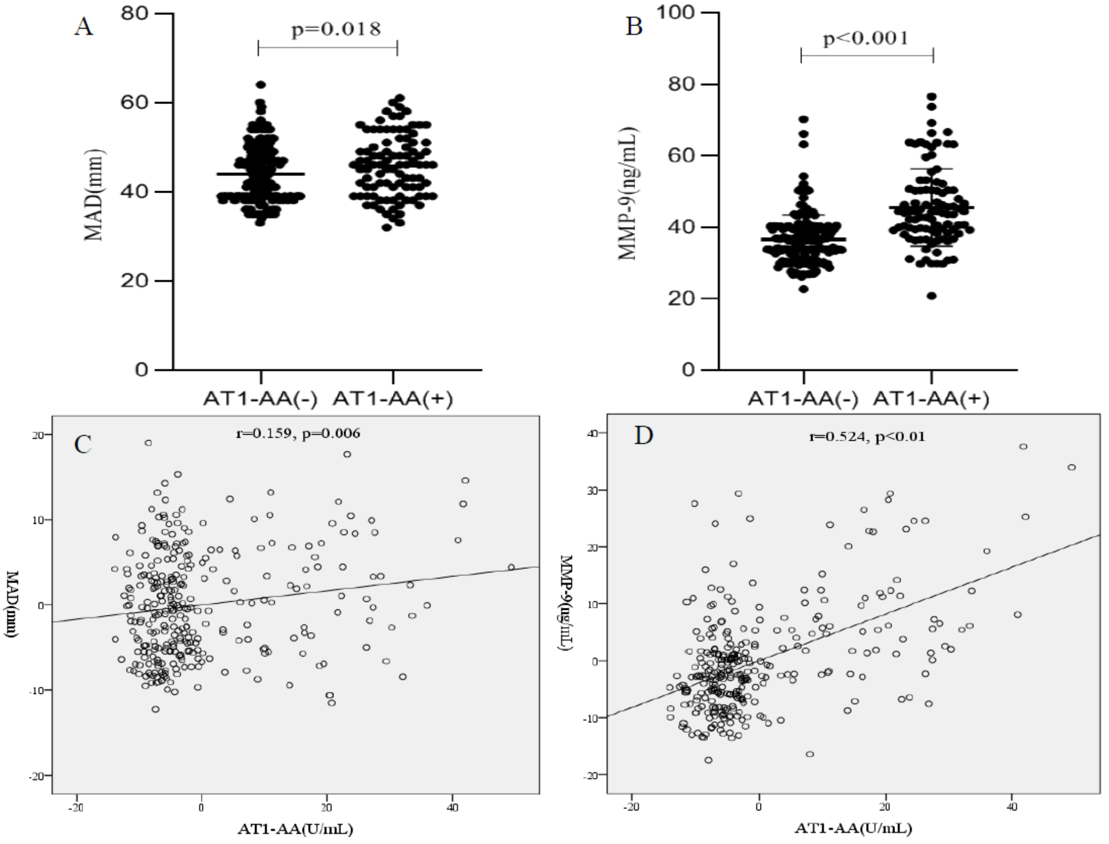

© 2021 Wu X et al. *JAMA Network Open*.

eTable. Multivariable Logistic Regression for Antibody Associated With MAD

Predictor	β-Coefficient	Standard Error	95% Confidence Interval	*p* Value
AT1-AA	0.083	0.030	0.024 to 0.142	.006
Age	0.027	0.029	-0.030 to 0.084	.355
Gender	-1.375	0.825	-2.999 to0.249	.097
hypertension	-0.201	0.877	-1.926 to1.525	.819
Type of AAD	2.968	0.756	1.454 to 4.480	<.001

Adjusted for age, sex, hypertension, diabetes, treatment method, MMP-9, BUN, D-dimer, cTnT, hs-CRP and IL-6.

© 2021 Wu X et al. *JAMA Network Open*.
